# Capsule impaction presenting as acute small bowel perforation: a case series

**DOI:** 10.1186/1752-1947-6-121

**Published:** 2012-05-03

**Authors:** Giovanni D De Palma, Stefania Masone, Marcello Persico, Saverio Siciliano, Francesca Salvatori, Francesco Maione, Dario Esposito, Giovanni Persico

**Affiliations:** 1 Dipartimento di Chirurgia Generale, Geriatrica ed Endoscopia Diagnostica ed Operativa, Centro di Eccellenza per l’Innovazione Tecnologica in Chirurgia, Università di Napoli Federico II, Medicina e Chirurgia, Naples, Italy

## Abstract

**Introduction:**

Perforation caused by capsule endoscopy impaction is extremely rare and, at present, only five cases of perforation from capsule endoscopy impaction are reported in the literature.

**Case presentation:**

We report here two cases of patients with undiagnosed small bowel stenosis presenting with acute perforation after capsule endoscopy. Strictures in the small bowel were likely the inciting mechanism leading to acute small bowel obstruction and subsequent distension and perforation above the capsule in the area of maximal serosal tension.

Case 1 was a 55-year-old Italian woman who underwent capsule endoscopy because of recurrent postprandial cramping pain and iron deficiency anemia, in the setting of negative imaging studies including an abdominal ultrasound, upper endoscopy, colonoscopy and small bowel follow-through radiograph. She developed a symptomatic bowel obstruction approximately 36 hours after ingestion of the capsule. Emergent surgery was performed to remove the capsule, which was impacted at a stenosis due to a previously undiagnosed ileal adenocarcinoma, leading to perforation.

Case 2 was a 60-year-old Italian man with recurrent episodes of abdominal pain and diarrhea who underwent capsule endoscopy after conventional modalities, including comprehensive blood and stool studies, computed tomography, an abdominal ultrasound, upper endoscopy, colonoscopy, barium enema and small bowel follow-through, were not diagnostic. Our patient developed abdominal distension, acute periumbilical pain, fever and leukocytosis 20 hours after capsule ingestion. Emergent surgery was performed to remove the capsule, which was impacted at a previously undiagnosed ileal Crohn’s stricture, leading to perforation.

**Conclusions:**

The present report shows that, although the risk of acute complication is very low, the patient should be informed of the risks involved in capsule endoscopy, including the need for emergency surgical exploration.

## Introduction

Small bowel capsule endoscopy (CE) has become a commonly performed diagnostic test in patients affected by various small bowel diseases.

Impaction of the video capsule in the small bowel is a potential serious complication of this procedure, occurring in 0.75% to 21% of cases as reported by previous studies; its incidence rate is greatly dependent on procedural indication and patient characteristics [1,2]. Small bowel perforation due to CE impaction is extremely rare and, to date, only five cases have been reported in the literature [3-7].

We report here two cases of patients with previously undiagnosed small bowel stenosis presenting with acute perforation as a result of capsule impaction.

## Case presentation

### Case 1

A 55-year-old Italian woman was referred to our division when she complained of severe cramping abdominal pain and vomiting approximately 36 hours after ingestion of a capsule endoscope.

**Figure 1:**
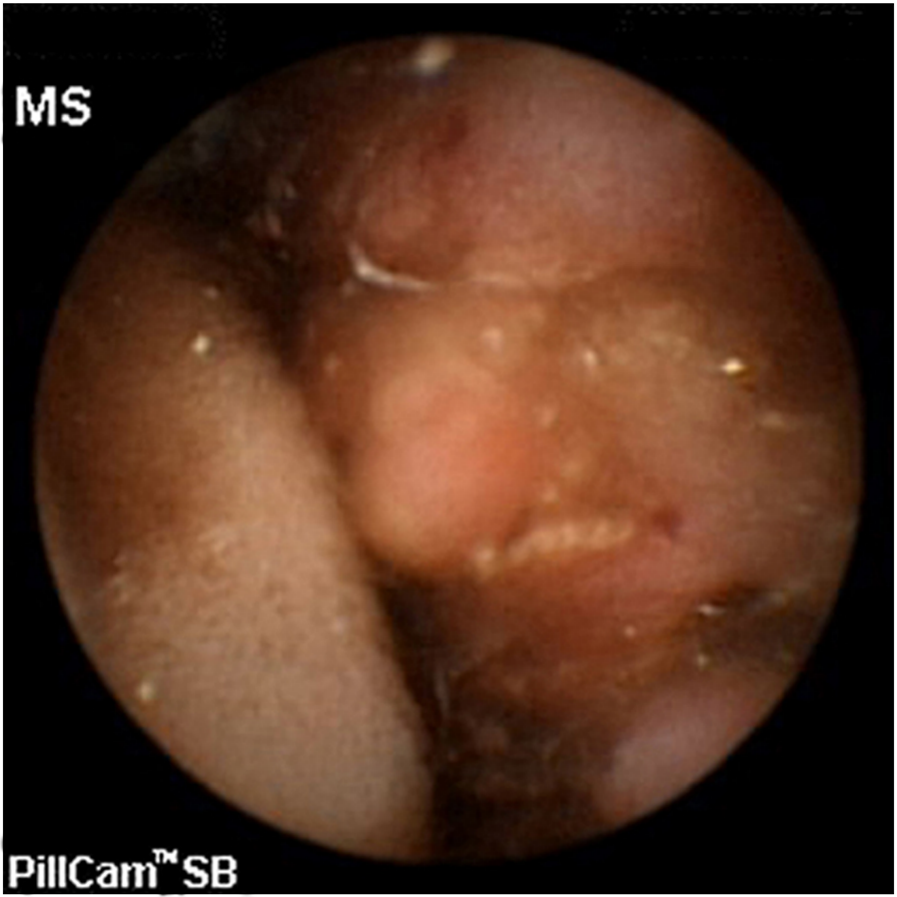
Capsule endoscopy image of a suspected neoplastic-like stricture in the mid-small bowel.

**Figure 2:**
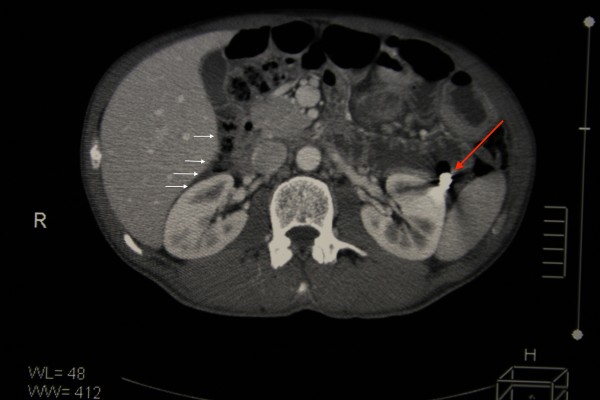
Computed tomography showing the capsule endoscope impacted in the ileum (red arrow) with extensive small bowel dilation along with free peritoneal air (white arrows).

**Figure 3:**
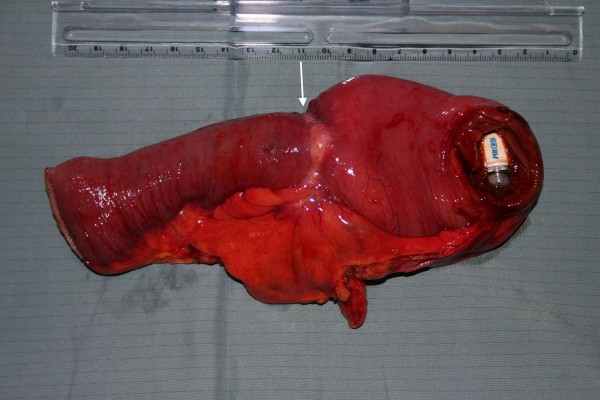
Resected small bowel segment with the constricting tumor (white arrow) and the video capsule.

Over the preceding years, she had undergone an extensive workup for recurrent postprandial cramping pain and iron deficiency anemia, including an abdominal ultrasound, upper endoscopy, colonoscopy and small bowel follow-through radiograph, all of which showed negative results. The physical examination on her admission revealed a mildly distended abdomen with increased bowel sounds. Laboratory tests showed normal results except for a red blood cell count of 3.15 million/mL (normal: 4.0 million to 5.4 million/mL) and hemoglobin of 9.8g/dL (normal: 12g/dL to 16g/dL). A plain abdominal film demonstrated a retained capsule in her small bowel without clear-cut signs of bowel obstruction. The endoscopist obtained images from the CE revealing a suspected neoplastic-like stricture in her mid-small bowel (Figure 1). Over the following 24 hours, our patient experienced progressive abdominal distention, worsening abdominal pain and vomiting, as well as development of leukocytosis. A computed tomography (CT) scan was then performed showing that the capsule was trapped in her proximal ileum, and that there was extensive small bowel dilatation along with free peritoneal air (Figure 2). Our patient underwent an urgent laparotomy during which an ileal perforation was found (Figure 3) proximal to a neoplastic-like stricture in the mid-ileum. An ileal resection with end-to-end anastomosis was therefore performed. Her postoperative course was uneventful, and our patient was discharged seven days later. Pathology confirmed the diagnosis of a well-differentiated ileal adenocarcinoma penetrating the entire wall and the mesentery, without lymph node invasion. Eleven months later, our patient had been treated with chemotherapy and was feeling generally well.

### Case 2

A 60-year-old Italian man arrived at our department with abdominal distension and acute periumbilical pain. Over the two preceding years, our patient had undergone an extensive negative workup performed for recurrent episodes of abdominal pain and diarrhea, including comprehensive blood and stool studies, CT, abdominal ultrasound, esophogastroduodenoscopy, colonoscopy, barium enema and small bowel follow-through. Twenty-four hours prior to his arrival, our patient had undergone CE, the results of which were still pending.

On admission, our patient was febrile. His physical examination revealed a distended abdomen with increased bowel sounds and marked tenderness in the right lower quadrant. Laboratory tests showed a white blood cell count of 16,300 cells/mL (normal: 4,500 to 10,500 cells/mL) and hemoglobin of 10.7g/dL (normal: 12g/dL to 16g/dL).

**Figure 4:**
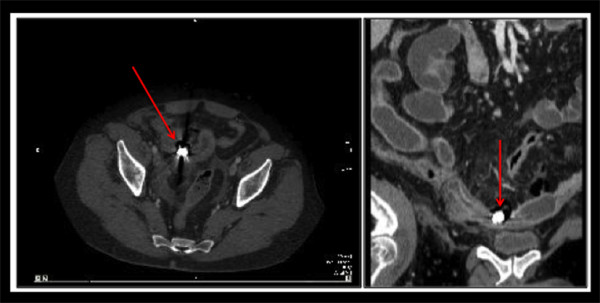
**Computed tomography scan of the abdomen in case 2.** Scan shows the presence of the capsule endoscope located in the distal ileum (red arrows), stricture of the small bowel along with extensive small bowel distension, a thickened bowel wall and loculated free air.

**Figure 5:**
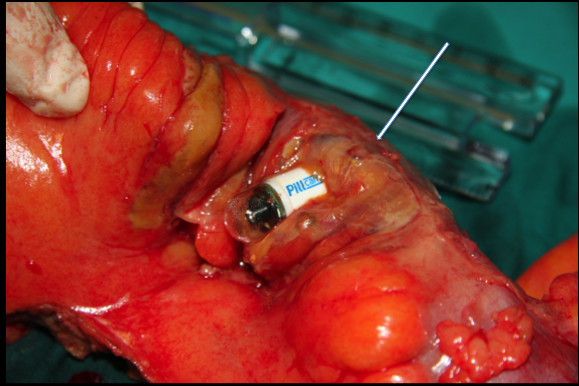
**Surgical specimen showing the stricture of the small bowel and the area of perforation (white arrow).** The capsule is taken out of a surgical incision in the terminal ileum after the resection.

A CT scan of his abdomen was performed and showed the presence of the capsule endoscope located in his distal ileum and a stricture along with extensive small bowel distension, a thickened bowel wall and loculated free air (Figure 4). Our patient underwent an urgent laparotomy. During the surgical exploration, an ileum perforation was noted proximal to an inflammatory-like stricture located in his distal ileum (Figure 5), which led to the resection of the inflamed segment with ileocolic anastomosis. Histology confirmed the diagnosis of Crohn’s disease. His postsurgical course was uneventful, and our patient was discharged 10 days later and treated subsequently with medications for Crohn’s disease with good results.

## Discussion

CE is a safe and well-tolerated procedure. To date, in clinical practice, the risk of capsule retention at the site of a previously unknown small bowel stricture remains the main complication of CE. The International Conference on Capsule Endoscopy consensus defined capsule impaction as retention of a capsule endoscope in the small bowel for a minimum of two weeks or as capsule retention in the bowel for a shorter period requiring medical, endoscopic or surgical intervention [8]. The frequency of capsule retention seems to be dependent mostly on the clinical indication for CE, ranging from 0% in healthy patients to 21% in patients with small bowel stricture. Larger single institution studies reported an impaction rate of up to 2.5% [1,2,9,10]. Capsule impaction usually occurs at sites of structural abnormality in the small bowel, usually ulcers, masses or strictures caused by Crohn’s disease, non-steroidal anti-inflammatory drugs use, radiation enteritis or surgical anastomosis.

In previously reported cases of capsule impaction, patients have generally been asymptomatic, even when the capsule has remained impacted for a very long time. However, some case reports described the possibility of acute obstruction or perforation due to capsule retention.

Patients with abdominal pain, distension and vomiting after CE should be evaluated for impaction and small bowel obstruction or perforation. The diagnosis may be aided by examination of capsule images, which can reveal an obstructing lesion, or show repetitive images of the same area of mucosa. Abdominal radiography or CT will typically demonstrate the retention of the capsule in the small bowel in association with signs of bowel distension or perforation.

To date, there have been five documented cases of small bowel perforation caused by an impacted capsule retention [3-7]. Perforation due to capsule retention occurred in four patients with Crohn’s disease and in one patient with visceral adhesions. Perforation happened from one day after ingestion of the capsule to two months after retention.

In the present cases, perforation occurred within 60 hours and 20 hours of the capsule ingestion in a patient with undiagnosed ileal adenocarcinoma and in a patient with undiagnosed Crohn’s disease, respectively. Strictures in the small bowel were likely the inciting mechanism leading to acute small bowel obstruction and subsequent distension and perforation above the capsule in the area of maximal serosal tension.

Our patients had no history of obstructive symptoms and both had undergone extensive diagnostic work up before the CE, including negative comprehensive blood, endoscopic and radiological studies. Our case series supports previous claims that barium studies have limited sensitivity for predicting capsule passage. CT or magnetic resonance imaging enteroclysis are being investigated as potential alternative screening methods, with promising results. There is, however, no accepted method to safely avoid capsule retention at present [11].

A newly designed non-visualizing dissolvable capsule (patency capsule: Given Imaging, Yoqneam, Israel) has been developed to minimize the risk of retention. This device starts its dissolution process 30 hours after ingestion even if one end of the capsule is impacted in a stricture. The patency capsule has been used as a screening test to assess the passage of an endoscopic capsule in patients at risk for small bowel strictures. There has been concern, however, about the efficacy and safety of this device because the patency capsule itself has also been shown to result in symptomatic small bowel obstruction in a few cases [12].

## Conclusions

In conclusion, although the risk of acute complication is very low, the patient should be informed of the risks involved in CE, including the potential need for emergency surgical exploration.

## Consent

Written informed consent was obtained from the patients for publication of this manuscript and accompanying images. Copies of the written consent are available for review by the Editor-in-Chief of this journal.

## Competing interests

The authors declare that they have no competing interests.

## Authors’ contributions

GDDP, MP, SM, SC, FM and GP have made substantial contributions to the conception, design, acquisition and interpretation of data; GP, SM, FM and DE performed the surgical interventions; FS and DE have been involved in drafting the manuscript and revising it critically for important intellectual content. All authors read and approved the final manuscript.
